# Protein sumoylation in normal and cancer stem cells

**DOI:** 10.3389/fmolb.2022.1095142

**Published:** 2022-12-19

**Authors:** Qiuhong Zhu, Panpan Liang, Cuiying Chu, Aili Zhang, Wenchao Zhou

**Affiliations:** ^1^ Intelligent Pathology Institute, The First Affiliated Hospital of USTC, Division of Life Sciences and Medicine, University of Science and Technology of China, Hefei, Anhui, China; ^2^ Department of Cardiovascular and Metabolic Sciences, Lerner Research Institute, Cleveland Clinic, Cleveland, OH, United States

**Keywords:** sumoylation, stem cell, ubiquitin-like protein, cancer stem cell (CSC), post-tranlational modifications

## Abstract

Stem cells with the capacity of self-renewal and differentiation play pivotal roles in normal tissues and malignant tumors. Whereas stem cells are supposed to be genetically identical to their non-stem cell counterparts, cell stemness is deliberately regulated by a dynamic network of molecular mechanisms. Reversible post-translational protein modifications (PTMs) are rapid and reversible non-genetic processes that regulate essentially all physiological and pathological process. Numerous studies have reported the involvement of post-translational protein modifications in the acquirement and maintenance of cell stemness. Recent studies underscore the importance of protein sumoylation, i.e., the covalent attachment of the small ubiquitin-like modifiers (SUMO), as a critical post-translational protein modification in the stem cell populations in development and tumorigenesis. In this review, we summarize the functions of protein sumoylation in different kinds of normal and cancer stem cells. In addition, we describe the upstream regulators and the downstream effectors of protein sumoylation associated with cell stemness. We also introduce the translational studies aiming at sumoylation to target stem cells for disease treatment. Finally, we propose future directions for sumoylation studies in stem cells.

## 1 Introduction

Post-translational protein modifications (PTMs), the reversible attachment of chemical groups to proteins, regulate multiple aspects of protein functions and thus play key roles in essentially all physiological and pathological processes. The stemness of normal and cancer stem cells, which is presented as the capacity of self-renewal and differentiation, is strictly under the control of a sophisticated network of PTMs. Recent studies underscore the importance of protein sumoylation, i.e., the covalent attachment of the small ubiquitin-like modifiers (SUMO), as a critical PTM in the stem cell populations in development and tumorigenesis (Bogachek et al., 2014; Bogachek et al., 2016; Du et al., 2016; Zhang et al., 2020a). In these stem cells, sumoylation functions to regulate several pivotal processes including protein stability, signal transduction, subcellular protein distribution, gene transcription, epigenetic profiles, and genome integrity.

The sumoylation machinery is composed of SUMO proteins, the SUMO-conjugating enzymes and the SUMO-deconjugating enzymes. SUMO proteins belong to the ubiquitin-related protein family and are conserved in essentially all eukaryotes (Fu et al., 2014). The diversity of the SUMO proteins seems to be increased during evolution. There is only one SUMO gene in *Drosophila melanogaster* and *Saccharomyces cerevisiae*, but at least four SUMO genes have been identified in vertebrates and flowering plants (Augustine and Vierstra, 2018). In mammals, SUMO proteins are classified into SUMO1 and SUMO2/3 categories that are conjugated to the lysine residuals of substrate proteins (Zhao, 2018). Similar to ubiquitination, SUMO conjugation to target proteins is executed by SUMO E1, E2 and E3 enzymes. In a classic scenario, the SUMO E1 enzymes SAE1/2 (SUMO-activating enzyme subunit 1/2) activate SUMO proteins and transfer SUMO to the sole E2 enzyme Ubc9, which in turn with the help of SUMO E3 enzymes PIAS1-4 (the protein inhibitor of activated STAT 1-4) transfers SUMO onto protein substrates (Gareau and Lima, 2010). On the other hand, SUMO proteins can be removed from substrates by a handful of desumoylation enzymes called sentrin-specific proteases (SENPs) (Gareau and Lima, 2010; Zhao, 2018).

Due to the indispensable functions of protein sumoylation, genetic mutations of the core components of the SUMO machinery rarely occur in mammalian cells (Seeler and Dejean, 2017). Sumoylation plays crucial roles in development. Associations between misregulated sumoylation and developmental defects have been demonstrated in plants, fruit flies, planarian, zebrafish, and frogs (Alkuraya et al., 2006; Nie et al., 2009; Li et al., 2012; Augustine and Vierstra, 2018; Thiruvalluvan et al., 2018; Bertke et al., 2019). In humans, it has been reported that protein sumoylation regulates craniofacial development (Pauws and Stanier, 2017). The situation of protein sumoylation is complicated in tumor biology. Upregulated expression of both sumoylation- and desumoylation-related enzymes has been detected in cancers (Seeler and Dejean, 2017). Meanwhile, sumoylation occurs on both oncogenes such as Myc and β-catenin and tumor suppressors such as p53, PTEN, and BRCA1 (Morris et al., 2009; Wu and Chiang, 2009; Bassi et al., 2013; Karami et al., 2017; Sun et al., 2018). Therefore, sumoylation plays a critical but complex role in tumors. This review will focus on the protein sumoylation in stem cells as the pivotal cell population in normal development and tumorigenesis.

## 2 Roles of sumoylation in normal and cancer stem cells

### 2.1 Embryonic stem cells

Embryonic stem cells (ESCs) are the pluripotent stem cell population existing in the very early stage of development. Sumoylation contributes to ESC maintenance through multiple mechanisms. The stemness of ESCs largely relies on a handful of transcription factors (Tsankov et al., 2015; Knaupp et al., 2017), whose activities could be controlled by sumoylation. For example, SUMO1 modification of the transcription factor Oct4 at lysine 118 stabilizes Oct4 protein to promote self-renewal of mouse ESCs (Zhang et al., 2007). The expression of proviruses and endogenous retroviruses (ERVs) is strictly repressed in ESCs to avoid insertional mutagenesis (Yang et al., 2015; Miles et al., 2017). A genome-wide siRNA screening revealed sumoylation factors *SUMO2*, *Ube2i*, *Sae1*, *Uba2* and *Senp6* as key determinants for provirus silencing in ESCs. Moreover, SUMO2-sumoylation of TRIM28 is necessary for its recruitment onto the proviral DNA, resulting in the deposition of the repressive H3K9me3 mark and the repression of ERVs (Yang et al., 2015), thereby maintaining the genomic integrity of ESCs.

Whereas several studies indicate the importance of sumoylation in maintaining stemness of ESCs, a few investigations suggest that sumoylation may function to keep ESCs from regaining totipotency. Proteomic analysis of mouse ESC revealed that SUMO2/3 primarily modified repressive chromatin complexes and thus prevented chromatin opening, impeding the conversion of ESC to 2-cell-embryo-like states (Theurillat et al., 2020). In addition, SUMO2 modification of the chromatin organizer SATB2 drove ESC differentiation in response to retinoic acid, which may be due to the rewiring of transcriptional networks and the chromatin interactome of ESCs (Antonio Urrutia et al., 2021). Furthermore, SUMO2/3 modification of the linker histone H1 facilitated its fixation onto ultra-condensed heterochromatin in ESCs, whereas loss of sumoylation de-compacted the chromatin and reactivated totipotency (Sheban et al., 2022).

### 2.2 Somatic stem cells and progenitor cells

Somatic stem cells as undifferentiated cells exist throughout animal bodies. With the potential to differentiate into specialized cell types, somatic stem cells function to maintain tissue homeostasis by replenishing dying cells and regenerating damaged tissues. The descendants of stem cells, named as progenitor cells, bear further reduced differentiation potency and only give rise to a specific type of cells (Gillich et al., 2020). Sumoylation is crucial for maintenance of both somatic stem cells and progenitor cells in multiple species. In adult Drosophila testis, reduction of sumoylation promoted differentiation of somatic cyst stem cells and impaired the proliferation of these cells (Lv et al., 2016). In Xenopus, high expression of the SUMO-conjugating enzyme Ubc9 is required for proliferation of retinal progenitors through regulation of cell cycle exit (Terada and Furukawa, 2010).

The importance of sumoylation is preserved in mammalian somatic stem cells and progenitor cells. Inducible knockout of Ubc9 in adult mice resulted in rapid disappearance of stem cells in the small intestine, leading to the depletion of the intestinal proliferative compartment (Demarque et al., 2011). Uterine stromal stem cells are activated and integrated into the regeneration area during menstruation, which is correlated with enhanced protein sumoylation in these CD34^+^KLF4^+^ stem cells (Yin et al., 2019). On the other side of the coin, desumoylation enzymes have critical roles in somatic stem cells. High expression of SENP2 is required for trophoblast proliferation and differentiation during placentation (Chiu et al., 2008; Jiang et al., 2011; Maruyama et al., 2016). In addition, SENP5 may play a role in the development of cardiac structures by deconjugation of SUMO1 (Zhang et al., 2020b). Furthermore, SENP1 promotes the migration and proliferation of adipose-derived stem cells (Wu et al., 2021).

### 2.3 Hematopoietic stem and progenitor cells

Hematopoietic stem and progenitor cells (HSPCs) with the capacity of self-renewal and differentiation into mature blood cell lineages are responsible for lifelong maintenance of hematopoiesis (Mende et al., 2022). Sumoylation regulates both maintenance and differentiation of HSPCs. HSPCs display higher SUMO contents than their differentiated progeny (Sahin et al., 2014). The sumoylation E1 enzyme SAE1 is essential for HSPC maintenance during fetal hematopoiesis in zebrafish (Li et al., 2012; Yuan et al., 2015). SUMO modification of lineage-specific transcriptional factors modulates myeloid progenitor proliferation and macrophage differentiation in chicken (Tillmanns et al., 2007). On the other side, the desumoylation enzyme SENP1 is required for erythropoiesis in liver in mouse (Yu et al., 2010).

### 2.4 Induced pluripotent stem cell

Adult somatic cells can be reprogrammed to pluripotent stem cells by forced expression of key transcription factors, leading to the generation of induced pluripotent stem cells (iPSC) that are similar to ESCs in many aspects (Kleiman and Engle, 2021). Sumoylation occurs on several reprogramming factors and thus regulates the formation of iPSCs. Sumoylation of KLF4 inhibits pluripotency induction of mouse fibroblasts into iPSCs (Tahmasebi et al., 2013). Likewise, reprogramming efficiency of the orphan nuclear receptor Nr5a2 is attenuated by sumoylation in induction of mouse iPSCs (Heng et al., 2010). Furthermore, comprehensive RNA interference screens reveal sumoylation as a major block of iPSC formation (Cheloufi et al., 2015; Borkent et al., 2016).

### 2.5 Cancer stem cells

Cancer stem cells (CSCs) are a small proportion of tumor cells that can self-renew and differentiate into other types of tumor cells. CSCs are believed to be the tumor initiating cells with strong resistance against therapies, thereby contributing to tumor initiation, progression, and relapse (Saygin et al., 2019). In addition, CSCs are acting as a key contributor to bypassing immunotherapy with immune checkpoint inhibitors (Rouzbahani et al., 2022). Altered global sumoylation has been observed in CSCs, but the functions of sumoylation in tumorigenesis may depend on the specific tumor types. Hypersumoylation has been reported to be a feature of glioma stem cells that is crucial for maintaining their tumorigenic capacity (Zhang et al., 2020a). In addition, sumoylation may drive the proneural to mesenchymal transition, a malignant phenotypic shift, in glioma stem cells (Chen et al., 2022). Moreover, a panel of inhibitors against SUMO E1 and E3 enzymes resulted in functional loss of CSCs in breast and colon cancers, indicating the requirement of sumoylation in CSC maintenance (Bogachek et al., 2016). While these studies indicate a tumor-supportive role of sumoylation in CSCs, some investigations demonstrate the tumor-repressive functions of sumoylation. In mice harboring a conditional ablation of *Apc* gene in intestinal stem cells or CSCs of intestinal cancer, deletion of a single allele of the sole SUMO E2 enzyme Ubc9 significantly increased the number of Lgr5 positive CSCs, which was accompanied by reduced global sumoylation levels in the polyps (Lopez et al., 2020). Of note, specific signaling pathways may be finely tuned by sumoylation to exert tumor-supportive or tumor-suppressive functions in CSCs. For example, the PML moiety of PML/RARA is sumoylated at the K160 site, which is required for efficient immortalization of primary hematopoietic progenitor cells and leukemic transformation (Zhu et al., 2005). On the other side, upon all-trans retinoic acid treatment, the orphan nuclear receptor TR2 associated with PML nuclear bodies becomes sumoylated and acts as a repressor for Oct4, a process in which sumoylation functions to suppress stemness (Gupta et al., 2008). Alternatively, sumoylation of different key proteins involved in a specific pathway may generate different outcomes and either support or suppress the stem cell phenotypes. The Wnt/β-catenin signaling pathway that is frequently dysregulated in CSCs provides a good example (Fan et al., 2022). Whereas numerous proteins within the Wnt/β-catenin pathway can be modified by sumoylation, sumoylation may positively or negatively regulate CSC self-renewal and the consequent occurrence, development, recurrence, and metastasis in different cancers (Fan et al., 2022).

**FIGURE 1:**
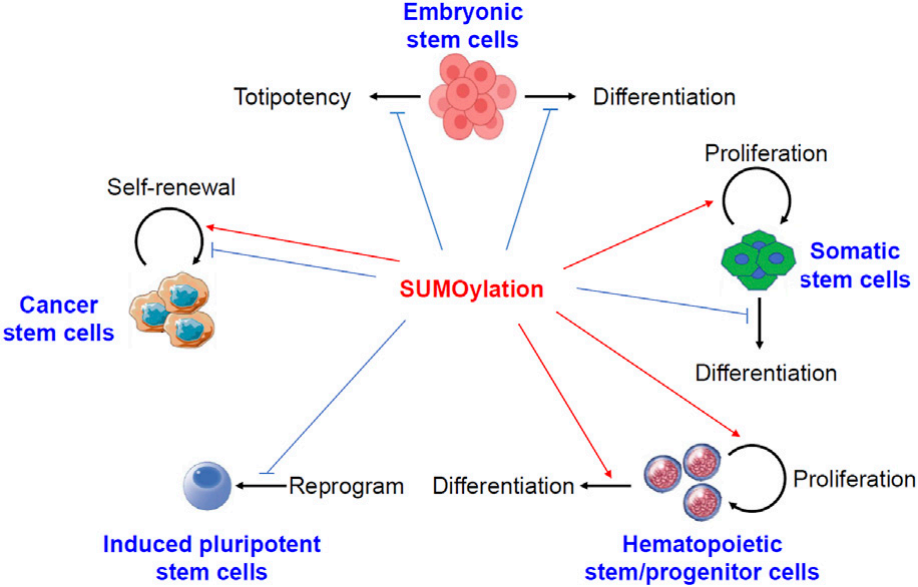
Pivotal roles of protein sumoylation in different kinds of stem cells. Sumoylation keeps embryonic stem cells in a stable state by preventing either differentiation or totipotency. In somatic stem cells, sumoylation promotes cell proliferation but prevents differentiation. In hematopoietic stem and progenitor cells, sumoylation activates both cell proliferation and differentiation. Sumoylation generally inhibits the formation of induced pluripotent stem cells. However, conflicting reports described both the activation and suppression of self-renewal of cancer stem cells by sumoylation of different substrates.

The roles of protein sumoylation in different kinds of stem cells have been summarized in Figure 1.

## 3 Sumoylation regulators in stem cells

### 3.1 The SUMO modifiers

The abundance of free SUMO proteins is a limiting factor that constrains the speed and frequency of global sumoylation. Consistently, expression of SUMO modifiers regulates protein sumoylation and hence the phenotypes of stem cells. In primary human adipose-derived stem cells, the age-dependent genome-wide alterations in chromatin accessibility are concurrent with altered SUMO protein expression under stress conditions (Shan et al., 2018). Meanwhile, forced overexpression of SUMO1 severely impairs viability of ESCs, which is likely ascribed to an excess accumulation of SUMO1-conjugated substrates (Lee et al., 2019). On the contrary, SUMO1 haploinsufficiency due to a chromosomal translocation in a human patient leads to cleft lip and palate, highlighting the requirement of SUMO1 modifiers and sumoylation of critical genes in palatogenesis, a process involved several types of cells including stem cells (Alkuraya et al., 2006). Therefore, stem cells may need an appropriate amount of free SUMO modifiers, and either excessive or insufficient SUMO expression would impact cell stemness.

### 3.2 The sumoylation enzymes

Conjugation of SUMO modifiers to substrate proteins are processed by a set of enzymatic machinery composed of E1, E2 and E3 enzymes (Gareau and Lima, 2010; Zhao, 2018). In line with the essential role of sumoylation in stem cells, components of the sumoylation machinery regulate multiple phenotypes of stem cells. Colorectal CSCs have higher SUMO E1 enzyme expression relative to non-CSCs, and genetic disruption or overexpression demonstrate a positive correlation between SUMO E1 levels and cancer cell stemness (Du et al., 2016). In addition, upregulation of the SUMO E2 enzyme Ubc9 is required for reprogramming of mouse embryonic fibroblasts into iPSCs (Tahmasebi et al., 2014). Ubc9 is also essential for ESC survival (Tahmasebi et al., 2014). When ESCs transit into pluripotent 2-cell-like cells, the SUMO E3 enzyme PIAS4 is down-regulated, which is sufficient to activate the transcriptional program for embryo development (Yan et al., 2019). These observations indicate that the sumoylation enzymes may promote cell stemness.

### 3.3 The desumoylation enzymes

SUMO modifiers could be removed from substrate proteins by a handful of desumoylation enzymes called SENPs (Gareau and Lima, 2010; Zhao, 2018). SENP2 is highly expressed in trophoblast cells and regulates trophoblast proliferation and differentiation (Chiu et al., 2008). In addition, postnatal loss of SENP6 in osteochondroprogenitor cells in the bone marrow causes premature aging, resulting in impaired skeletal development in mice (Li et al., 2018). However, these studies ascribe the functions of SENPs to some pivotal molecular targets such as the p53 protein, suggesting that the outcome of desumoylation may largely depend on the modified substrates. Therefore, the role of global desumoylation in the maintenance of cell stemness remains unclear.

### 3.4 Other regulatory proteins

In addition to the abovementioned classic components of the sumoylation machinery, multiple regulatory proteins have been reported to mediate sumoylation. Recent studies have discovered some atypical SUMO E3 ligases in stem cells. CBX4, a member of the Polycomb Repressive Complex 1 (PRC1) that transcriptionally represses downstream genes during development, has E3 SUMO ligase activity (Wu et al., 2022). CBX4 promotes sumoylation and accumulation of BMI1 (Ismail et al., 2012), a transcriptional repressor with essential roles in the self-renewal of many normal and cancer stem cells (Sangiorgi and Capecchi, 2008; Chen et al., 2017). Likewise, a ubiquitin E3 ligase UHRF2 also acts as a SUMO E3 ligase (Oh and Chung, 2013). UHRF2 expression promotes organoid formation from primary intestinal adenomas, suggesting an oncogenic role of UHRF2 in CSCs. UHRF2 may sumoylate the Wnt pathway effector Tcf4 to maintain hyperactive Wnt signaling in CSCs (Li et al., 2020). Similarly, the Tripartite Motif-Containing Protein 28 (TRIM28) with the SUMO E3 ligase activity binds to the lncRNA PVT-1 and sumoylates the phosphatidylinositol 3-kinase catalytic subunit type 3 (Vps34), which enhances the ubiquitination and degradation of the tumor suppressor complex 2 (TSC2), thus contributing to stem cell phenotypes such as invasion in osteosarcoma (Tsang et al., 2022). In addition to atypical enzymes, regulatory proteins may indirectly participate in sumoylation process in stem cells. The peptidyl-prolyl cis-trans isomerase Pin1 promotes global sumoylation and maintenance of glioma stem cell (Zhang et al., 2020a). The isomerase activity of Pin1 is required for sumoylation, suggesting that Pin1 may alter protein configuration to facilitate the interaction between sumoylation enzymes and substrates (Zhang et al., 2020a). Increased expression of the tumor suppressor protein ARF delays age-associated stem cell exhaustion, suggesting a role of ARF in maintaining normal stem cells (Carrasco-Garcia et al., 2017). Despite the lack of enzymatic activity, ARF has been found to enhance PIAS1 sumoylation and suppress PIAS1 activity (Alagu et al., 2018), which may be ascribed to the association between ARF and the SUMO E2 enzyme Ubc9 (Wang et al., 2015). Along with the increasing interest in sumoylation and stem cells, future studies would certainly reveal more unconventional regulators of sumoylation with essential roles in normal and cancer stem cells.

## 4 Molecular processes under the control of sumoylation in stem cells

### 4.1 Protein stability

As the ubiquitin-like proteins, SUMO modifiers share many structural similarities with ubiquitin. It is not surprising to see the crossover of these two kinds of PTMs in regulating protein stability. Poly-sumoylation often serves as a signal for the recruitment of SUMO-targeted ubiquitin ligases (STUbLs), resulting in the subsequent poly-ubiquitination on a neighboring lysine residue on the substrate protein, which finally leads to proteasomal degradation (Sriramachandran et al., 2019; Keiten-Schmitz et al., 2020). Sumoylation-mediated degradation of suppressive regulators would activate some critical pathways required for the maintenance of cell stemness. In leukemic stem cells, sumoylation of the beta-catenin antagonist CBY1 reduces its stability through the ubiquitin-proteasome system, which contributes to the activation of beta-catenin signaling and the resistance against tyrosine kinase inhibitors (Mancini et al., 2015). Of note, sumoylation may also function to stabilize substrate proteins. SUMO modifiers may compete with ubiquitin for the same lysine residues and thus inhibit ubiquitination (Desterro et al., 1998). In addition, sumoylated proteins may have strong affinity to ubiquitination inhibitors that prevent the addition of poly-ubiquitin chain (Sha et al., 2019). Several studies have reported the SUMO-mediated stabilization of master transcription factors that are crucial for the maintenance of cell stemness. The transcription factor Oct4 is a key regulator in ESC, CSC, and iPSC. Sumoylation of Oct4 at lysine 118 increases the protein stability, DNA binding affinity, and transcriptional activity of Oct4 (Wei et al., 2007; Zhang et al., 2007). Interestingly, reproductive toxic cobalt and nickel metals induce Oct4 sumoylation and stabilize Oct4 protein in a concentration-dependent manner, indicating a dynamic control of Oct4 by sumoylation (Yao et al., 2014). Sumoylation also promotes the stability of SALL4, which interacts with Oct4 in promoting cell stemness (Yang et al., 2012).

### 4.2 Protein interaction

Like other PTMs, sumoylation could facilitate the non-covalent interaction between proteins. Such interaction is often mediated by binding of SUMO interaction motifs (SIMs) to SUMO modifiers. A typical SIM is composed of a core of hydrophobic residues flanked by negatively charged amino acids (Liang et al., 2021). Interaction between two proteins can be enhanced by the binding of multiple SIMs to SUMO modifiers (Liang et al., 2021). However, it is not sure whether SIM is indispensable for the SUMO-mediated protein interactions. Sumoylation-mediated protein interactions play important roles in maintaining cell stemness. Recent studies have demonstrated the crucial roles of PML proteins in different kinds of stem cells (Zhou and Bao, 2014). Sumoylation of PML protein facilitates its interaction with the stem cell transcription factor c-Myc, which promotes the maintenance of glioma stem cells (Zhou et al., 2015; Zhang et al., 2020a). As sumoylation of PML proteins is prerequisite for the formation of PML nuclear bodies that function as hot-spots for the docking of several SIM-bearing proteins (Zhou and Bao, 2014), sumoylation may enhance the interaction between PML and different downstream effectors in different stem cells. In addition, sumoylation of OTUB2, a deubiquitinase participating in the maintenance of several CSCs, enables its interaction with transcriptional regulators YAP/TAZ to activate YAP/TAZ signaling and promote cancer cell stemness (Zhang et al., 2019). Interestingly, some studies show that sumoylation may perturb protein interactions. For example, it has been reported that sumoylation of either Oct4 or Sox2 impairs the interaction between the two proteins, although the impact on cell stemness remains unclear (Wu et al., 2012).

### 4.3 Protein localization

Sumoylation has the capacity to affect the subcellular localization of proteins and therefore regulate protein functions. The unsumoylated testicular receptor 2 (TR2) is localized to the PML nuclear bodies and functions as a transcriptional activator of Oct4 (Gupta et al., 2008). Sumoylation of TR2 releases it from the nuclear bodies and switches it into a repressor. In this scenario, sumoylation of TR2 enables the fine-tuning of Oct4 expression and regulates stem cell proliferation (Park et al., 2007). The Notch pathway plays key roles in regulating stem cells during development and tumorigenesis (Majumder et al., 2021). The Notch intracellular domain (NICD1) generated from cleavage of the Notch receptor is required for the transcriptional activation of Notch target genes. NICD1 is sumoylated in a stress-inducible manner, which enhances its nuclear localization and facilitates the recruitment of histone deacetylase 4 (HDAC4), thereby suppressing Notch target gene transcription (Antila et al., 2018). Given that sumoylation alters the interaction between proteins, localization of sumoylated proteins may to some extent depend on their interacting partners.

### 4.4 Epigenetic regulation

Epigenetic status to a large degree determines the state of stem cells. However, it was not until recently that people realized the importance of sumoylation in epigenetic regulations. Sumoylation is linked to specified cell fates. Suppressing sumoylation in ESCs promotes their conversion into 2C-like cells (2-cell-stage embryo). In this case, SUMO functions on heterochromatin to maintain proper H3K9me3 levels genome-wide and thus silences differentiation (Cossec et al., 2018). Further studies demonstrate that SUMO2/3 modification of the linker histone H1 facilitates its fixation onto ultra-condensed heterochromatin in ESCs, whereas disruption of sumoylation de-compacts the chromatin and evicts H1 to reactivate totipotency (Sheban et al., 2022). Moreover, SENP3-mediated desumoylation of RbBP5 protein, a regulatory component of the histone-modifying SET1/MLL complexes, facilitates H3K4 methylation and activates downstream gene transcription to dictate osteogenic differentiation of human stem cells (Nayak et al., 2014). Future studies may reveal more functions of sumoylation on epigenetic features including chromatin accessibility, DNA and histone modifications, and even RNA processing.

### 4.5 Transcriptional activation

Cell stemness is largely under the control of certain transcription factors that are frequently sumoylated. SUMO modifications may increase transcriptional activities of transcription factors. For example, sumoylation of the Yamanaka factor Oct4 augments its activity and promotes G1/S progression of murine ESCs (Wei et al., 2007; Campbell and Rudnicki, 2013). Likewise, sumoylation of ERalpha elevates its downstream gene transcription and activates proliferative signaling in murine uterine stem cells (Yin et al., 2019). On the other hand, sumoylation may negatively regulate the activities of transcription factors. GATA-1 is required for erythropoiesis, but sumoylation reduces its binding to the promoters of target genes (Yu et al., 2010). Similarly, sumoylation of Eya1, a conserved regulator of organ-specific stem cells, inhibits the downstream transcription (Sun et al., 2015). Moreover, SUMO modification negatively regulates the transcriptional activity of DPPA2, a critical transcription factor in mouse ESCs and embryo development (Yan et al., 2019). Besides transcription factors, sumoylation of transcriptional co-factors may also affect gene transcription. For instance, sumoylation of Nab protein, the coregulator of the transcription factor Krox20 that regulates hindbrain development, represses transcriptional activity of Krox20 (Garcia-Gutierrez et al., 2011).

### 4.6 Genome integrity

Faithful preservation of genome integrity is critical for the maintenance of self-renewal stem cells. Endogenous retroviruses (ERVs) and exogenous proviruses pose substantial threats to genome stability of ESCs (Xiang and Liang, 2021). Systematic siRNA screen revealed that sumoylation factors are among the key determinants for the establishment of provirus silencing in ESCs (Yang et al., 2015). Moreover, sumoylation facilitates the interaction between the lysine methyltransferase SETDB1 and the co-repressor KAP1 that function together to deposit H3K9me3 and suppress retrotransposition of ERVs (Thompson et al., 2015). Proper repair of DNA damages is another key process required for genome integrity. In differentiating mouse ESCs, sumoylation of thymine DNA glycosylase suppresses DNA strand-break accumulation and is essential for neural lineage commitment (Steinacher et al., 2019). Taken together, these findings highlight the importance of sumoylation in maintaining genome stability and genetic fidelity of stem cells.

**FIGURE 2:**
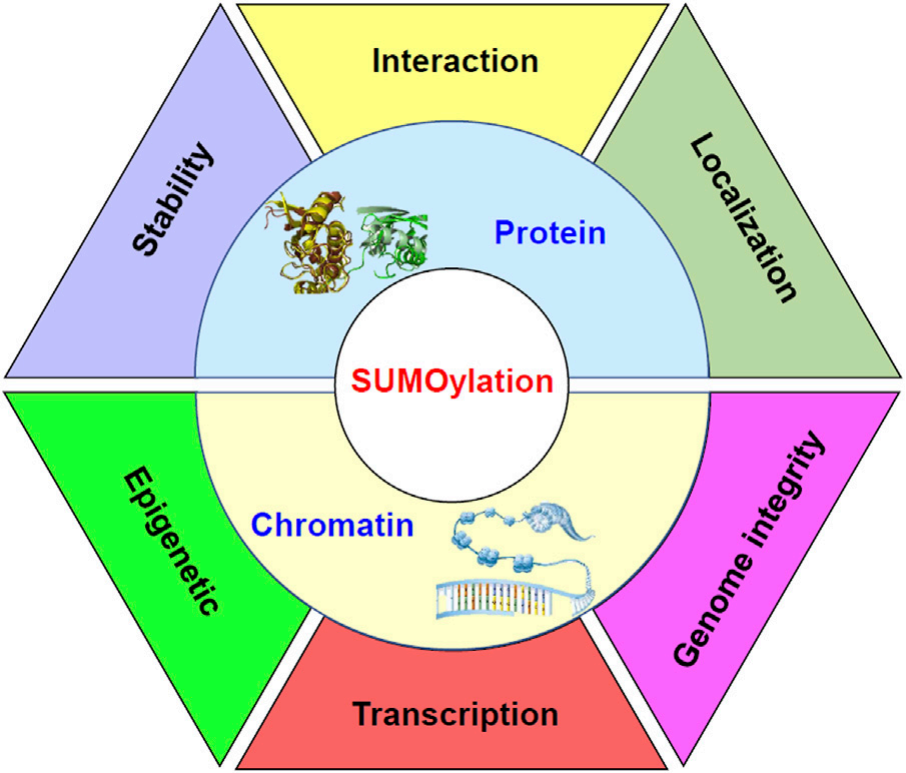
Diagram to demonstrate the molecular processes under the control of sumoylation in stem cells. Sumoylation regulates the stability, interaction, and localization of proteins. Meanwhile, sumoylation at chromatin may function as epigenetic signals. Sumoylation of transcription factors may affect the formation of the transcriptional machinery on the chromatin and regulate transcription of target genes. Furthermore, sumoylation of chromatin components may be important for the integrity of the genome.

Molecular processes under the control of sumoylation in stem cells have been summarized in Figure 2.

## 5 Sumoylation-related therapies targeting stem cells for disease treatment

In line with the close relationship between sumoylation and cell stemness, several studies have pointed out that manipulating sumoylation in normal and cancer stem cells may benefit disease treatment. When engrafted into brains, neural stem cells (NSC) not only differentiate to cellular replacements of mature neural cell types but also modulate inflammation and angiogenesis (Baker et al., 2019). Thus NSCs are promising therapeutic tools for the treatment of central nervous system (CNS) diseases including epilepsy, stroke, and neural degenerative diseases (Baker et al., 2019). When global sumoylation in NSCs is elevated by overexpressing the SUMO E2-conjugase Ubc9, NSCs are endowed with stronger resistance against deprivation of oxygen/glucose, resulting in increased survival and neuronal differentiation in ischemic lesions in mouse brains (Bernstock et al., 2019). Therefore, sumoylation may be a critical target to optimize the effectiveness of exogenous NSC medicines in ischemic stroke and probably other CNS diseases.

Most sumoylation-related therapeutic studies are focusing on CSCs. Fukuda et al. (2009) found that ginkgolic acid and its structural analog anacardic acid inhibited protein sumoylation *in vitro* and *in vivo* by blocking the formation of the E1-SUMO intermediate. After then, ginkgolic acid and anacardic acid had been shown to reduce cell invasiveness and impair CSCs in basal breast cancer and colorectal cancer through disturbing the SUMO-conjugated form of the Transcription Factor AP-2-Alpha (TFAP2A) (Bogachek et al., 2016; Kharkar, 2017). In glioma stem cells, melatonin attenuates SUMO1 sumoylation and disturbs interaction between nestin (NES) and c-Myc, resulting in reduction of c-Myc levels and inhibition of stemness (Lee et al., 2018). In hepatocellular carcinoma, dexamethasone reduces sumoylation and accumulation of HIF1alpha, Oct4 and other proteins, thereby disrupting CSCs to improve chemotherapy (Jiang et al., 2020). Moreover, a natural product compound McM25044 extracted from actinomycete has the capacity to selectively inhibit CSCs by direct targeting the SAE1/2 complex to impede the sumoylation cascade. McM25044 treatment of patient-derived breast, colorectal and leukemic CSCs reduces global sumoylation and impairs cell stemness *in vitro* and *in vivo*. Meanwhile, McM25044 shows negligible effects on normal stem cells (Benoit et al., 2021). These discoveries underscore the potential of inhibiting sumoylation to combat CSCs.

Interestingly, on some proteins sumoylation could act both as a glue to facilitate binding of downstream signaling molecules and as a trigger to initiate subsequent polyubiquitination and proteasomal degradation. If so, either stimulating or inhibiting sumoylation may have anti-tumor effects but *via* different pathways. This is well exemplified in PML proteins in leukemia and glioblastoma. Sumoylation of PML/RARA is required for immortalization of primary hematopoietic progenitor cells and leukemic transformation (Zhu et al., 2005), suggesting that inhibiting sumoylation may prevent leukemia. However, arsenic trioxide treatment promotes sumoylation of PML/RARA oncoprotein and the consequent ubiquitin-mediated proteasomal degradation, thereby acting as a cure for promyelocytic leukemia (Jeanne et al., 2010). Likewise, arsenic trioxide causes degradation of PML protein, leading to the destabilization of the PML-interacting c-Myc protein and the inhibition of glioma stem cells (Zhou et al., 2015). On the other side, juglone treatment inhibits Pin1-mediated sumoylation of PML protein and impedes the binding of c-Myc to PML, which severely impacts stemness of cancer but not normal stem cells (Zhang et al., 2020a). These facts further highlight protein sumoylation as a pivotal target for disrupting CSCs to treat cancers.

## 6 Conclusion and perspectives

Sumoylation often acts as molecular switch triggering the functional shift of signaling networks in stem cells. Sumoylation of key factors often determines the fate of normal stem cells. Many facts have shown the ability of sumoylation to prevent differentiation of stem cells. However, sumoylation may also initiate differentiation of pluripotent cells. The chromatin organizer SATB2 is sumoylated upon retinoic acid treatment, which promotes SATB2 binding at differentiation genes, leading to the rewiring of transcriptional networks of ESCs and the transition of pluripotency to differentiation (Antonio Urrutia et al., 2021). Sumoylation may govern the direction of differentiation. SUMO modification of the transcription co-factor myocardin (MYOCD) in pluripotent fibroblasts strongly activates the expression of cardiogenic genes, leading to a switch from smooth muscle to cardiac muscle differentiation (Wang et al., 2007). In CSCs, key regulators may function either as an oncogene or tumor repressor depending on the sumoylation status. The orphan receptor TR2 activates Oct4 to enhance embryonal carcinoma cell proliferation when unsumoylated. Yet sumoylation of TR2 alters its nuclear localization and interacting co-regulator, switching TR2 from an activator to a repressor (Park et al., 2007). Likewise, the PML protein has been recognized as a tumor suppressor for a long time (Wang et al., 2017). But recent studies have revealed the oncogenic roles of the sumoylated PML in CSCs (Jeanne et al., 2010; Zhou et al., 2015).

The difference between SUMO1 and SUMO2/3 categories may have a role in the switch on/off process. The amount and distribution of SUMO1- and SUMO2/3-modified proteins vary during mouse brain development. SUMO2/3-modified proteins accumulate in neural progenitor cells, whereas higher SUMO1-sumoylation is detected in mature neurons (Hasegawa et al., 2014). In line with these discoveries, SUMO1 but not SUMO2 overexpression is not tolerated in murine ESCs, suggesting a negative role of SUMO1-sumoylation in embryonic cells (Lee et al., 2019). However, SUMO1- rather than SUMO2/3-sumoylation seems to promote the maintenance of CSCs. Melatonin suppresses glioma initiating cells by reducing SUMO1 but not SUMO2/3 modifications (Lee et al., 2018). Likewise, higher SUMO1-sumoylation is observed in glioma stem cells, whereas similar SUMO2/3-sumoylation levels are detected in stem and non-stem tumor cells in glioblastoma (Zhang et al., 2020a). Interestingly, the desumoylation enzymes may also participate in mediating the signaling from SUMO1 and SUMO2/3 modifiers. For example, SENP1 with a strong desumoylation activity towards SUMO1 rather than SUMO2/3 has an essential role in mouse embryonic development (Sharma et al., 2013). Taken together, these studies strongly support the distinct roles of SUMO1 and SUMO2/3 modifiers in cell stemness. Aside from the different categories of SUMO conjugates, the length of the conjugates (poly versus mono sumoylation) and the numbers of modifications (single versus multiple sumoylation) may have regulatory roles in the functional SUMO switch.

The in-depth study of sumoylation-regulated stemness requires a comprehensive understanding of the unique sumoylated proteins in stem cells. This remains a challenge because of the low abundance of endogenous sumoylated proteins, urging the development of sensitive identification strategies. Mass spectrometry has been popular so far for proteomic study of global sumoylation. Meanwhile, different methods have been utilized to enrich sumoylated protein for mass spectrometry analysis. SUMO conjugates could be purified with anti-SUMO antibodies or SUMO affinity trap with multiple SIMs (Lopitz-Otsoa et al., 2019; Li et al., 2021; Pronot et al., 2021). Ectopic epitope-tagged SUMO proteins are often expressed to further facilitate enrichment of sumoylated proteins (Barroso-Gomila et al., 2021). For identification of sumoylation sites on the sumoylated proteins, an artificial tryptic site may be introduced into the ectopic SUMO protein to shorten the SUMO chains. This is often achieved by substitution of a specific residue with an arginine in the SUMO protein, which will generate a signature peptide with a di-glycine remnant attached to the lysine residue on substrate proteins after tryptic digestion. The strategy has been used to analyze sumoylation in human iPSCs, resulting in identification of 976 sumoylation sites on 427 substrates (Mojsa et al., 2021). However, ectopic overexpression of SUMO proteins would elevate global sumoylation levels. Moreover, point mutation of SUMO protein may prevent the formation of poly-SUMO chain. Therefore, the identified sumoylated proteins must be carefully validated for their involvement in maintenance of stemness.

The past decade has seen a growing interest in the role of sumoylation in regulating cell stemness. Future studies will surely provide a better understanding of the upstream regulatory mechanisms and the downstream effector pathways of sumoylation, which would not only broaden our understanding of the essential roles of sumoylation in normal development and tumorigenic progression, but also open the way to novel molecular interventions and development of new therapeutics.

## Glossary

### The following abbreviations are used in this document:

PTMs: post-translational protein modifications
SUMO: small ubiquitin-like modifiers
SAE1/2: SUMO-activating enzyme subunit 1/2
PIAS1-4: protein inhibitor of activated STAT 1-4
SENPs: sentrin-specific proteases
ESCs: embryonic stem cells
ERVs: endogenous retroviruses
TRIM28: Tripartite Motif-Containing Protein 28
Ubc9: Ubiquitin-Conjugating Enzyme E2I
HSPCs: Hematopoietic stem and progenitor cells
iPSC: induced pluripotent stem cells
KLF4: Kruppel Like Factor 4
Nr5a2: Nuclear Receptor Subfamily 5 Group A Member 2
Lgr5: Leucine-Rich Repeat-Containing G-Protein Coupled Receptor 5
PML: Promyelocytic Leukemia Protein
RARA: retinoic acid receptor alpha
TR2: Testicular Receptor 2
CBX4: Chromobox 4
PRC1: Polycomb Repressive Complex 1
BMI1: a polycomb group (PcG) protein
UHRF2: Ubiquitin-Like With PHD And Ring Finger Domains 2
Tcf4: Transcription Factor 4
PVT-1: Plasmacytoma Variant Translocation-1
Vps34: phosphatidylinositol 3-kinase catalytic subunit type 3
TSC2: tumor suppressor complex 2
Pin1: Peptidyl-Prolyl Cis-Trans Isomerase NIMA-Interacting 1
ARF: Alternative Reading Frame
STUbLs: SUMO-targeted ubiquitin ligases
CBY1: Chibby Family Member 1
SALL4: Spalt Like Transcription Factor 4
SIMs: SUMO interaction motifs
OTUB2: OTU Domain-Containing Ubiquitin Aldehyde-Binding Protein 2
YAP: Yes1 Associated Transcriptional Regulator
TAZ: Tafazzin, Phospholipid-Lysophospholipid Transacylase
NICD1: Notch intracellular domain
HDAC4: histone deacetylase 4
RbBP5: Retinoblastoma-Binding Protein 5
SET1: SET Domain Containing 1A, Histone Lysine Methyltransferase
MLL: Lysine Methyltransferase 2A
Eya1: Eyes Absent Homolog 1
DPPA2: Developmental Pluripotency Associated 2
Krox20: Early Growth Response 2
SETDB1: SET Domain Bifurcated Histone Lysine Methyltransferase 1
KAP1: KRAB [Kruppel-Associated Box Domain]-Associated Protein 1, also known as tripartite motif-containing protein 28, TRIM28
NSC: neural stem cells
CNS: central nervous system
SATB2: Special AT-Rich Sequence-Binding Protein 2
GATA-1: GATA-Binding Protein 1 (Globin Transcription Factor 1)
Nab: NGFI-A Binding Protein (EGR1 Binding Protein)
MYOCD: myocardin
NES: nestin
TFAP2A: Transcription Factor AP-2-Alpha
